# Social Support and Frailty in HIV Positive and Negative Men Who Have Sex With Men

**DOI:** 10.1093/geroni/igaa057.3009

**Published:** 2020-12-16

**Authors:** Laura Graf, Mackey Friedman, Steven Meanley, James Egan, Andre Brown, Deanna Ware, Michael Plankey, Sabina Haberlen

**Affiliations:** 1 Johns Hopkins Bloomberg School of Public Health, Brooklyn, New York, United States; 2 University of Pittsburgh, Pittsburgh, Pennsylvania, United States; 3 University of Pennsylvania, Philadelphia, Pennsylvania, United States; 4 Georgetown University Medical Center, Washington, District of Columbia, United States; 5 Johns Hopkins University, Baltimore, Maryland, United States

## Abstract

Social support is linked to a myriad of positive health outcomes, yet little is understood about its potential role on frailty development among older men who have sex with men (MSM). We evaluated data from 929 MSM aged 40-81 years enrolled in the MACS Health Aging sub-study. Social support (Social Provisions Scale[SPS-24]; range 24-96) was high, though slightly lower among the HIV-positive versus HIV-negative men (median: 80 vs. 82, p=0.12). Each SD increase in social support associated with a 21% decrease in incident frailty (Fried phenotype), independent of age, race, and education (aIRR=0.79, IQR[0.65, 0.97]), though attenuated after adjustment for depressive symptoms. This protective association was observed to be strongest among HIV-positive MSM. High social support is a strength among older MSM, which associates with positive frailty outcomes. Assessing and strengthening social support systems may have potential as a psychosocial component of frailty interventions.

